# Supplementary material for: New heterodont odontocetes from the Oligocene Pysht Formation in Washington State, U.S.A., and a reevaluation of Simocetidae (Cetacea, Odontoceti)
Source: PeerJ. 2023 Jun 23;11:e15576. doi: 10.7717/peerj.15576 (PMC10292202; doi:10.7717/peerj.15576)
Supplement: Supplemental Information 2 — Character/states were all treated as unordered in this study and follow the morphological character list from Albright et al. (2018) and Boessenecker et al. (2020). For this work, two characters were modified, and four new ones were added (highlighted in bold) for a total of 338 characters. [file peerj-11-15576-s002.docx]

**Supplemental File 2. List of character/states used in the phylogenetic analysis.**

Character/states were all treated as unordered in this study and follow the morphological character list from Albright et al. (2018) and Boessenecker et al. (2020). For this work, two characters were modified, and four new ones were added (highlighted in bold) for a total of 338 characters.

1. Baleen: Absent (0); present (1) (Miller, 1923).

2. Rostrum: Narrows in width anteriorly or anterior half approximately the same width as posterior half (0); anterior part widened transversely (1) (Muizon, 1994).

3. Length of rostral portion of maxilla: Short, rostral portion of maxilla < 43% of condylobasal length excluding the premaxillae (0); intermediate, rostral portion between 48 and 70% of modified condylobasal length (1); elongate, rostral portion > 73% modified condylobasal length (2) (modified from Barnes, 1985).

4. Anterior half of maxilla: Its lateral edge in cross section forms an angle of 60° to 45° (0); highly acute angle with flattened maxilla (1) (modified from Barnes, 1990).

5. Vomerine trough, or mesorostral canal: Open, vomer in cross section is V-shaped or U-shaped (0); partially or completely filled in with bone, becomes solid rod of bone (1) (Moore, 1968).

6. Rostral constriction well anterior to antorbital notch: Absent (0); present (1) (Barnes, 1985).

7. Width of rostrum at antorbital notch: Wide, rostral width > 92% the width across of middle of orbits (0); fairly wide, between 82% and 72% the width across orbits (1); narrow, between 68% and 46% the orbital width (2); very narrow, between 32% and 29% the orbital width (3).

8. Premaxilla in dorsal view: Portion adjacent to and anterior to nasal opening narrows or remains the same width anteriorly (0); widens at anterior end (1).

9. Premaxillae on anterior third of rostrum: With skull in dorsal view, contact and fused along midline (0); contact but not fused along midline (1); sporadic contact along midline (2); separated by a narrow fissure for entire length (3); clear separation although mesorostral canal still has a partial roof (4); very wide separation, mesorostral canal is completely open along entire length (5) (modified from Muizon, 1988; Fordyce, 1994; Messenger & McGuire, 1998). If the morphology varies within a single individual, code based on the morphology present for the majority of the anterior third of the rostrum.

10. Premaxillae on middle third of rostrum: With skull in dorsal view, contact and fused along midline (0); contact but not fused along midline (1); sporadic contact along midline (2); separated by a narrow fissure for entire length (3); clear separation although mesorostral canal still has a partial roof (4); very wide separation, mesorostral canal is completely open along entire length (5) (modified from Muizon, 1988; Fordyce, 1994; Messenger & McGuire, 1998). If the morphology varies within a single individual, code based on the morphology present for the majority of the anterior third of the rostrum.

11. Suture between maxilla and premaxilla on rostrum: Suture fused along most of rostrum (0); anterior quarter of rostrum fused with remaining portions unfused (1); unfused along entire rostrum but articulation tight (2); suture is unfused and marked by a deep grooved (3) (modified from Fordyce, 1994; Messenger & McGuire, 1998).

12. Posterior region of rostral edge: Lateral margin is straight or gently concave with skull in dorsal view (0); slightly bowed outward causing a V-shaped antorbital notch (1); bowed far outward forming a deep U-shaped antorbital notch (2); lateral margin of maxilla nearly contact lacrimal and jugal resulting in the opening of the notch being a narrow slit (3).

13. Posterodorsal portion of maxilla: Sutured to frontal (0); not sutured, separated from frontal by a distinct gap, which is situated between the maxilla anterodorsal and the frontal ventrally (1) (McLeod et al., 1993).

14. Steep face on anterolateral edge of zygomatic process of maxilla clearly separating it from rostral portion of maxilla: Absent (0); present but low (1); present and well developed (2).

15. Posterior end of ascending process of maxilla: Tapers to a point (0); end is squared-off (1). Cannot be scored for taxa with maxilla covering most of supraorbital processes of the frontal.

16. Posterior wall of antorbital notch: Maxilla (0); lacrimal and jugal (1).

17. Palatal surface of rostrum: Flat or gently concave (0); bears pronounced longitudinal keel along the midline of the rostrum (1) (McLeod et al., 1993). The keel is formed by vomer and medial edges of maxillae.

18. Palatal surface of maxilla: Bears few vascular foramina, those that are present are small (0); bears many, large vascular foramina that open laterally and anterolaterally into long sulci (1); bears numerous small vascular foramina that lack sulci (2).

19. Posterior end of palatal surface of rostrum at the suture between palatine and maxilla: Concave to flat, depth of rostrum, measured as the dorsoventral distance from the level of the lateral edge of rostrum to the ventral-most part of rostrum, is < 8% the width of rostrum at antorbital notches (0); convex, depth between 11% and 25% the rostral width (1); highly convex, rostral depth > 27% the rostral width (2). The convexity of the rostrum is a part of characters included by Barnes (1985) and Fordyce (1994).

20. Palatine: Sutured to maxilla and suture visible (0); fused to maxilla (1) (Messenger & McGuire, 1998).

21. Palatine/Maxilla suture: In ventral view, suture between both palatines and both maxillae is straight transversely or bowed anteriorly (0); maxillae have posterior processes that separate palatines anteriorly, suture around midline is V-shaped and points posteriorly (1).

22. Teeth in females: Erupt in adulthood (0); do not erupt in adulthood but remain in the crypt (1) (Moore, 1968).

23. Tooth rows: Separated and diverge posteriorly (0); left and right sides adjacent to the midline and thus close together, are nearly parallel (1) (Zhou, 1982).

24. Number of double-rooted teeth in maxilla: None (0); 1 or 2 (1); 4 (2); 5 (3); 6 (4); 7 (5); 8 or more (6). The primitive state for Cetacea and Artiodactyla is probably state “3”; however, the character was coded with no teeth as state “0” in order to implement the ordering of states in the phylogenetic analyses. Muizon (1987, 1991, 1994) noted that the absence of all double-rooted teeth is a synapomorphy of Platanistidae plus Squalodelphinidae.

25. Number of teeth with alveoli completely enclosed in the maxilla: None, 1, 2, or 3 (0); 7 to 8 (1); 9 to 10 (2); 11 to 13 (3); 15 to 17 (4); 21 to 23 (5); 26 to 29 (6); 32 to 39 (7); 50 to 60 (8) (modified from Messenger & McGuire, 1998). State “1” is probably the primitive condition for Cetacea and Artiodactyla; however, the character was coded with no teeth as state “0” in order to implement the ordering of states in the phylogenetic analyses. Barnes (1985) listed extreme polydonty as a synapomorphy of the genus *Parapontoporia*.

26. Large diastemata between posterior buccal teeth: Absent (0); present (1).

27. Tooth enamel: Smooth (0); bears reticulating striae (1); nodular (2) (Zhou, 1982).

28. Lower anterior mandibular teeth: Conical (0); spatulate (1); laterally compressed (2) (Messenger & McGuire, 1998). Heyning (1989b) described state “1” and Moore (1968) described state “2”.

29. Lower anterior teeth: Deeply rooted with at least half of tooth forming root (0); not deeply rooted (1) (Flower, 1872; Moore, 1968).

30. Swelling on lingual side of posterior lower teeth: Present (0); absent (1) (Flower, 1867; Messenger & McGuire, 1998).

31. Posterior buccal teeth: High peg-shaped teeth, crown base is < 70% the crown height (0); nearly an equilateral triangle, crown base is between 100% to 148% the crown height (1); wide low teeth, crown base is > 180% the crown height (2).

32. Buccal teeth ectocingulum: Absent (0); present (1).

33. Buccal teeth entocingulum: Present (0); absent (1).

34. Buccal teeth: Bear accessory cusps (0); cusps absent (1) (Kellogg, 1923b).

35. Central cusp as compared to denticles: Much larger (0); subequal (1). Cannot be scored for taxa that lack denticles.

36. Anterior-most mandibular teeth: Oriented anteriorly (0); vertical (1); inclined posteriorly (2) (Moore, 1968; Messenger & McGuire, 1998).

37. Anterior-most mandibular teeth: Smaller than posterior teeth (0); approximately same size as posterior teeth (1); greatly enlarged (2) (modified from Flower, 1872; Heyning, 1989b; Muizon, 1991; Messenger & McGuire, 1998).

38. Number of teeth number in lower jaw: None (0); 1 (1); 2 (2); 8 to 10 (3); 11 to 12 (4); 13 to 14 (5); 19 to 23 (6); 24 to 27 (7); 28 to 34 (8); more than 40 teeth (9) (modified from Messenger & McGuire, 1998). State “3” is probably the primitive condition for Cetacea and Artiodactyla; however, the character was coded with no teeth as state “0” in order to implement the ordering of states in the phylogenetic analyses.

39. Mandible in lateral view: Straight (0); arched dorsally (1) (McLeod et al., 1993).

40. Length of mandibular symphysis: Short, mandibular symphysis forms less than 28% of the total mandibular length (0); long, symphysis length between 33% and 40% of the mandibular length (1); very long, symphysis forms more than 48% of the length of the mandibles (2) (modified from Heyning, 1989b; Barnes, 1990).

41. Mandibular symphysis: At least one third of the symphysis is fused (0); sutured but unfused (1); not sutured, shallow longitudinal fossa for fibrocartilaginous connection (2); not sutured, well-developed longitudinal groove for fibrocartilaginous connection (3) (Fordyce, 1994; Fitzgerald, 2006). Barnes (1990) listed a loose mandibular symphysis as a synapomorphy of Mysticeti. State “1” is probably the primitive condition for Cetacea and Artiodactyla; however, the fused condition was designated as state “0” in order to implement the ordering of states in the phylogenetic analyses.

42. Longitudinal groove on underside of mandible: Absent (0); present (1) (Miller, 1923).

43. Mandible: Bowed medially (0); straight (1); slightly bowed laterally, a line drawn from the posteriormost to anteriormost points stays within body of mandible (2); strongly bowed outward, line from anterior to posterior points does not entirely lie within body of mandible (3) (Miller, 1923; Sanders & Barnes, 2002).

44. Mandibular fossa: Small or absent (0); present and large, forms a large cavity posterior to mandibular foramen (1) (Barnes, 1990).

45. Shape of coronoid process: Long and low, height of mandible at coronoid process < 89% the length of coronoid (0); height of mandible between 100% and 177% the length of coronoid (1); short and high, height between 203% and 300% coronoid length (2); very high and short, height > 450% the length of coronoid (3).

46. Dorsal surface of condyle: Elevated above dorsal edge of the rest of mandible, not counting coronoid process (0); at same level as rest of mandible (1) (Sanders & Barnes, 2002).

47. Supraorbital processes of frontal: Are horizontal or gradually slope lateroventrally away from vertex of skull (0); abruptly depressed at base to a level noticeably below that of dorsal surface of interorbital region (1); slope laterodorsally away from vertex (2) (Miller, 1923; Messenger & McGuire, 1998).

48. Dorsal edge of orbit relative to lateral edge of rostrum: Below the level of the edge of rostrum (0); orbit low, either in line with edge of rostrum or slightly above it, height of orbit < 46% the height of rostral base, both heights measured relative to the lateral edge of rostrum (1); orbit low, height of dorsal edge of orbit between 50% and 92% the rostral height (2); orbit high, height between 100% and 128% the rostral height (3); orbit elevated well above rostrum, orbital height > 163% the rostral height (4).

49. Frontal/Maxilla suture: With skull in lateral view, suture is approximately horizontal, and lateral exposure of frontal over the orbit does not thicken posteriorly (0); angled posterodorsally at and angle of 50° to 70° from axis of rostrum, lateral exposure of frontal thickens posteriorly (1) (Miller, 1923). This character cannot be scored for taxa that lack overlap of the maxilla onto the frontal.

50. Anterior edge of the supraorbital process: Oriented anteromedially (0); oriented slightly anterolaterally, forms an angle < 30° with sagittal plane (1); oriented anterolaterally, forms an angle between 35° and 60° (2); oriented anterolaterally or laterally, forms and angle between 68° and 90° degrees (3); oriented posterolaterally, forms an angle between 107° and 120° (4); oriented posterolaterally, forms an angle > 142° (5). The anterior edge of the supraorbital process participates in the formation of the antorbital notch. For this character supraorbital process includes frontal, lacrimal, and/or jugal, whichever bone forms the posterolateral wall of the notch. An antorbital notch opening anteriorly was listed by Barnes (1990) as a synapomorphy of Odontoceti.

51. Lacrimal: Forms small bone on anterior edge of orbit with small orbital portion (0); enlarged both posteromedially and anterolaterally paralleling anterior edge of supraorbital process of frontal, shaped like a thick rod (1).

52. Lacrimal: Restricted below supraorbital process of frontal (0); wraps around anterior edge of supraorbital process of frontal and slightly overlies its anterior end (1); greatly expanded posterodorsally and covering much of lateral side of supraorbital process of frontal (2) (modified from Miller, 1923; Kellogg, 1923a).

53. Lacrimal foramen or groove: Present (0); absent (1).

54. Lacrimal and jugal: Separate (0); fused (1) (Miller, 1923; Heyning, 1989b).

55. Jugal and lacrimal: Jugal and lacrimal contact each other externally (0); lacrimal excluded from edge of skull, jugal directly contacts anterior edge of frontal (1) (modified from Miller, 1923).

56. Combined anteroposterior length of the lacrimal and jugal exposure that is posterior to antorbital notch: With skull in ventral view, exposure is small and combined length forms < 31% of anteroposterior distance from antorbital notch to postorbital ridge (0); intermediate, forms between 35% and 59% of that distance (1); large, forms between 62% and 69% that distance (2); very large, forms > 77% of that distance (3). The postorbital ridge is a curved ridge that extends from the postorbital process to the orbital foramen (Fordyce, 2002). It follows much of the course of the orbital nerve and demarcates the separation between the orbital and postorbital regions of the skull.

57. Jugal: Thick and sturdy (0); thin splint or incomplete or absent (1) (Miller, 1923).

58. Dorsolateral edge of proximal foramen of infraorbital canal: Formed by maxilla (0); formed by maxilla and lacrimal and/or jugal (1); formed by lacrimal and/or jugal (2); formed by frontal (3) (modified from Miller, 1923).

59. Ventromedial edge of proximal foramen of infraorbital foramen: Formed by maxilla (0); formed by maxilla and palatine and/or pterygoid (1); formed by palatine and/or pterygoid (2) (modified from Miller, 1923).

60. Maxillary infraorbital plate: Absent (0); present but small (1); present and large (2) (Miller, 1923). The infraorbital plate of the maxillary is the posterior part of the maxilla underlying the orbit.

61. Anteriormost point on the posterior edge of the supraorbital process: The anteriormost point is at the lateral edge of postorbital process (0); located laterally, between 70% and 74% of transverse distance from sagittal plane to the lateral edge of postorbital process (1); positioned approximately midway, located between 42% and 61% of that distance (2); medially positioned, located at a point < 34% of that distance (3).

62. Postorbital process: Long and projects posterolaterally and slightly ventrally (0); short and directed ventrally (1).

63. Infratemporal crest (Postorbital ridge): Present, forms well-defined curved ridge on posterior edge of sulcus for optic nerve (0); no well-defined ridge, region is gently convex (1).

64. Facial region of skull, skull in lateral view: Concave (0); flat (1); moderately arched dorsally (2); greatly arched dorsally (3) (Miller, 1923; Heyning, 1989b; Messenger & McGuire, 1998).

65. Anterior cluster of dorsal infraorbital foramina: Single (0); two (1); three or more (2) (Barnes, 1984). The anterior cluster does not include the one or two foramina that are in a far posterior position over the orbits.

66. Rostral basin: Absent or poorly defined (0); present, situated medial to antorbital notch and anterior to supraorbital process of frontal, best developed medially and ventrally where lateral edge of maxilla is very thin (1). The rostral basin is not a depression centered on the median plane, instead it is a bilateral structure.

67. Transverse distance between lateral edges of right and left premaxillae at antorbital notches: Small, distance < 48% the width of rostrum at antorbital notches (0); intermediate, distance between 52% and 64% the antorbital width (1); wide, distance > than 78% the antorbital width (2).

68. Premaxillae immediately anterior to external bony nares: Widely separate with skull in dorsal view, gap between medial edges of premaxillae > 63% the maximum width of external bony nares (0); narrow separation, gap between premaxillae between 56% and 32% the width of external nares (1); separation absent or nearly so, gap < 28% the nares width (2).

69. Premaxillae anterior to nasal openings: Are flat or concave, form a premaxillary sac fossa (spiracular plate) (0); convex transversely (1); form distinct bosses or “premaxillary eminences” with steep posterior faces on anterior edges of nasal openings (2) (Muizon, 1988; Barnes, 1990). This character was split into two characters by Messenger & McGuire (1998). They are combined here because a taxon with bosses cannot simultaneously have a fossa.

70. Premaxillary foramina: Absent (0); present and one on right side (1); two on right side (2); three on right side (3) (modified from Barnes, 1990).

71. Premaxillary foramen size: Right and left subequal (0); left larger than right (1); left much larger than right (2) (modified from Messenger & McGuire, 1998).

72. Position of premaxillary foramen: Far anterior of antorbital notch and anterior edge of supraorbital process (0); approximately medial to or posterior to antorbital notch region, which is at the junction of supraorbital process with rostrum (1).

73. Posterolateral sulcus from premaxillary foramen: Sulcus very short or absent (0); present and short (1); present and extends to level equivalent to middle of nasal openings (2) (modified from Muizon, 1988).

74. Premaxillae: Restricted to medial position adjacent to mesorostral canal and nasal opening (0); extended laterally covering much of the supraorbital process (1). The maxilla in turn overlies the premaxilla (Kellogg, 1923a).

75. Posteriormost end of nasal process of premaxilla: Located far anterior to anterior edge of supraorbital process/antorbital notch (0); just anterior to or in a transverse line with anterior edge of supraorbital process of the frontal (1); in line with anterior half of supraorbital process of frontal or halfway point, anteroposteriorly, of supraorbital process (2); in line with posterior half of supraorbital process or postorbital process of frontal (3); in line with gap between postorbital process and anterior tip of zygomatic process of the squamosal or in line with anterior tip of the latter process (4); in line with space between anterior tip of zygomatic process of squamosal and anterior edge of floor of the squamosal fossa or in line with anterior edge of floor of the squamosal fossa (5); located posterior to anterior edge of floor of the squamosal fossa (6).

76. Maxillary foramen (posteriormost dorsal infraorbital foramen): Absent (0); present and one, situated over supraorbital process of frontal (1); two (2); foramina absent because roof of canal that carries posterior branches of internal maxillary artery and the maxillary division of infraorbital nerve is unossified (3) (modified from Barnes, 1990). The maxillary foramen (or foramina) is distinct from the anterior cluster of dorsal infraorbital foramina.

77. Maxilla: Abuts anterior edge of supraorbital process of frontal or terminates anterior to it (0); partially covers supraorbital process (1); covers almost entire surface (2) (Miller, 1923; Fordyce, 1994). Fordyce erected state “1” for *Archaeodelphis* (Allen, 1921).

78. Posteriormost edge of the ascending process of maxilla: Situated well anterior to anterior edge of orbit (0); in transverse line with anterior half of supraorbital process of frontal or in line with the halfway point, anteroposteriorly, of supraorbital process (1); in line with posterior half of supraorbital process or in line with postorbital process of frontal (2); in line with gap between postorbital process and the anterior tip of zygomatic process of squamosal or in line with anterior tip of the latter process (3); in line with space between anterior tip of zygomatic process of squamosal and anterior edge of floor of squamosal fossa or in line with anterior edge of the floor of squamosal fossa (4); posterior to anterior edge of floor of squamosal fossa (5).

79. Anterolateral corner of maxilla overlying supraorbital process of frontal: Thin and equal in thickness to parts posteromedial (0); thickened with thinner maxilla in posteromedial direction (1). The thickened maxilla plus other nearby elevated structures (e.g. nasal opening, supraoccipital), delimit the edges of a broad fossa for the insertion of the maxillonasolabialis muscle.

80. Maxillary ridge: Absent (0); present (1); form transversely compressed and high crest (2); crest arches over and encloses a cavity for the melon (4) (modified from Miller, 1923; Muizon, 1987). Messenger & McGuire (1998) split this into two characters; however, this results in scoring the absence of the crests twice.

81. Anterior edge of nasals: In transverse line with incisors, canines, or intervening diastema (0); in line with P1 (1); in line with P2 or about 18% of the total rostral length towards anterior edge of rostrum (2); just anterior to or in line with anterior edge of supraorbital process of frontal (3); in line with anterior half of supraorbital process of frontal or in line with the halfway point, anteroposteriorly, of supraorbital process (4); in line with posterior half of supraorbital process or in line with postorbital process of frontal (5); in line with gap between postorbital process and the anterior tip of zygomatic process of squamosal or in line with the anterior tip of the latter process (6); in line with space between the anterior tip of zygomatic process of squamosal and anterior edge of the floor of squamosal fossa or in line with anterior edge of the floor of squamosal fossa (7); posterior to the anterior edge of the floor of the squamosal fossa (8).

82. Anterior edge of external bony nares: V-shaped, premaxillae gradually converge anteriorly to the midline (0); U-shaped, premaxillae abruptly converge anteriorly to the midline (1) (Muizon, 1988). Muizon (1988) described state “0” as heart-shaped.

83. Maxillae: In region anterior to nasal openings, maxillae are exposed lateral to premaxillae (0); maxillae are exposed at posterior end of roof of mesorostral gutter, medial to the premaxillae and nearly converge on midline (1); same as 1 except maxilla also exposed on anterior edge of external bony nares (2) (Muizon, 1988).

84. Ossicles: Absent (0); present, occur in anteromedial corners of external bony nares, probably a derivative of the maxilla (1) (Muizon, 1988).

85. Right premaxilla: Posterior edge approximately in line with posterior edge of left premaxilla (0); right premaxilla extended distinctly farther than left (1); right extended much farther than left (2) (modified from Barnes, 1990; Messenger & McGuire, 1998).

86. Transverse width of right premaxilla immediately anterior to external bony nares: Distinctly narrower than left premaxilla (0); subequal, width of right premaxilla within 10% of the width of left premaxilla (1); right wider, width is between 130% and 145% the width of the left (2); right much wider, width > 167% the width of the left (3).

87. Right premaxilla: Portion posterior to nasal openings wider than portion anterior to opening, with nasal septum angled anteriorly and to the right (0); portion anterior wider than portion posterior to nasal opening, septum angled anteriorly and to the left (1). Cannot be scored for taxa that lack extension of the premaxilla posterior to the nasal openings or those that lack asymmetrical widening of the right premaxilla.

88. External bony nares: Left and right are the same size (0); left is twice or more the size of the right (1) (Barnes, 1990).

89. Supracranial basin: Absent (0); present (1) (Heyning, 1989b).

90. Posterior end of premaxilla: Posterior end adjacent to lateral edge of nasal opening (0); angled slightly laterally resulting in the following sequence, from lateral to medial, in one transverse plane: premaxilla, maxilla, anterior edge of nasals or mesethmoid (1) (Muizon, 1988; Heyning, 1989b).

91. Angle of premaxillae anterior to external bony nares, skull in lateral view: Low angle, premaxillae form an angle < 28° with the lateral edge of rostrum (0); intermediate angle, form an angle between 30° and 40° (1); high angle, form an angle > 45° (2) (modified from Moore, 1968).

92. Premaxillae adjacent to nasal opening: Thin dorsoventrally and porous internally (0); pachyostotic, in direction perpendicular to face, and pachyosteosclerotic but nasals and premaxillae equally project dorsally and anteriorly (1); extreme pachyostosis, premaxillae adjacent to nasals project farther outward, anteriorly and dorsally (2) (modified from Moore, 1968).

93. Proximal ethmoid region: Not visible in dorsal view, roofed over by nasals (0); exposed dorsally (1) (Miller, 1923).

94. Mesethmoid: Forms T-shaped bone with median plate separating right and left nasal passages, not all of the dorsal part is divided by median plate (0); bears expanded posterodorsal plate which is not divided by median plate, median plate situated more ventrally (1) (modified from Muizon, 1984; Muizon, 1988). This bone may include parts of the cribriform and perpendicular plates of the ethmoid.

95. Shape of soft tissue external nares: Crescent with apices pointed anteriorly (0); crescent with apices pointed posteriorly, might be skewed (1); rectangular (2); a longitudinal slit, might be slightly sigmoidal or angled (3); comma-shaped (4) (modified from Messenger & McGuire, 1998).

96. Soft tissue nasal passages distal to bony external nares: Separate with two separate soft tissue external nares (0); separate for most of length but confluent just proximal to blowhole (1); confluent (2) (Heyning, 1989b; Messenger & McGuire, 1998).

97. Orientation of right soft tissue nasal passages: Oriented anterodorsally (0); oriented dorsally (1) (Messenger & McGuire, 1998). Cannot be scored for taxa that have little soft tissue in the facial area. In those taxa the bony and soft tissue external nares are in the same position.

98. Right posterior dorsal bursa: Small (0); hypertrophied to form the spermaceti organ (1) (Cranford et al., 1996).

99. Melon: Absent (0); small (1); forms large sac that occupies much of the face (2); hypertrophied, forms a dome-shaped face (3) (Heyning & Mead, 1990).

100. Distal sacs: Absent (0); present, situated immediately distal to museau de singe (1) (modified from Heyning, 1989b). The vestibular sacs are considered homologous to the distal sacs of the right nasal passage of physeterids.

101. Left and right distal sacs: Equal in size (0); right larger, includes physeterid condition of no distal sac on the left nasal passage (1) (Mead, 1975; Heyning, 1989b).

102. Blowhole ligament: Absent (0); present (1) (Heyning, 1989b).

103. Right posterior nasal sac: Present and elongate, in some cases it reaches osteological vertex (0); reduced and short, never reaches osteological vertex (1); absent (2). This is the same as the frontal sac of physeterids.

104. Nasofrontal sacs: Absent (0); portions posterior to nasal passage present (1); same as previous state except that sacs extend anterodorsally around, and then in front of, the nasal passages; in dorsal view both nasofrontal sacs form a horseshoe shape (2) (Heyning, 1989b).

105. Inferior vestibule: Absent (0); present, forms a diverticulum of nasal passages posterior to blowhole ligament (1) (Heyning, 1989b).

106. Premaxillary sacs: Absent (0); present, form the most proximal diverticulum of nasal passages; they extend anteriorly adjacent to dorsal surfaces of premaxillae (1) (Heyning, 1989b).

107. Accessory sac: Absent (0); present, forms a small diverticulum of inferior vestibule and extends anterolaterally around the attachment of blowhole ligament to premaxilla (1) (Mead, 1975).

108. Inflection of nasal process of premaxilla: Absent, lateral edge of premaxilla is fairly straight or smoothly curved (0); present, the lateral edge of the posterior end of the premaxilla is sigmoidal with an anterior splint of maxilla that emarginates the posterior edge of premaxilla and splits it into a posterolateral plate and posteromedial splint (1) (derived from Fordyce, 1994).

109. Position of inflection of premaxilla: In transverse line with P1 (0); in line with P2 or about 18% of total rostral length towards anterior edge of rostrum (1); just anterior to or in line with anterior edge of supraorbital process of frontal (2); in line with anterior half of supraorbital process of frontal or in line with the halfway point, anteroposteriorly, of supraorbital process (3); in line with posterior half of supraorbital process or in line with postorbital process of frontal (4); in line with gap between postorbital process and the anterior tip of zygomatic process of squamosal or in line with the anterior tip of the latter process, space is absent in some taxa (5); in line with space between the anterior tip of zygomatic process of squamosal and anterior edge of the floor of squamosal fossa or in line with anterior edge of the floor of squamosal fossa (6); posterior to anterior edge of the floor of squamosal fossa (7). The position of the inflection cannot be scored for taxa that lack one.

110. Premaxillary cleft: Absent (0); present, posterior part of nasal process of premaxilla bears a distinct cleft that originates from at posterior edge of the premaxilla and continues anteriorly dividing the premaxilla in two (1); present but cleft is shallow (2). In odontocetes, the cleft begins at the anterior splint of the maxilla and helps divide the posterolateral plate from the posteromedial splint.

111. Premaxillae adjacent to and at posterior edge of the nasal opening: Do not clearly overhang maxillae (0); premaxillae overhang maxillae (1); premaxillae greatly enlarged laterally, region between lateral edge of the right premaxilla and supraoccipital is partially enclosed (2) (Muizon, 1991).

112. Narial pit: Absent (0); present, an anterior extension of nasal passage and forms a blind pocket in the maxilla just dorsal to maxilla/palatine suture; medial wall of pocket is formed by vomer (1). This character is usually not visible in complete skulls.

113. Posterior end of nasal process of premaxillae: In addition to facing at least partially dorsally, face anterolaterally (0); face anteriorly (1); face anteromedially (2) (modified from Moore, 1968).

114. Nasal bones: Two (0); one (1); none (2) (Heyning, 1989b).

115. Suture between right and left nasals and right and left frontals: Shifted towards right side (0); situated on midline (1); shifted towards left side (2) (Barnes, 1985). Character state “1” is probably the primitive condition for Cetacea and Artiodactyla; however, the character was coded with suture on the right side as state “0” in order to implement the ordering of states in the phylogenetic analyses.

116. Dorsoventral thickness of anterior edge of nasal: Very thin, nasal thickness < 82% of the anterior nasal width (0); thick, nasal thickness between 100% and 173% of the nasal width (1); very thick, > 200% the nasal width (2). Measurement taken approximately five millimeters from anterior edge.

117. Both nasals in dorsal view: Anterior edges are straight in one transverse plane (0); with point on midline and a gap on each side between premaxilla and nasal (1) (Moore, 1968). The shape of the nasals is based on the dorsal surface. In some cases the nasals may come to a point medially but at a more ventral position adjacent to the mesethmoid plate (state 0) (e.g. *Inia* AMNH 93412).

118. Nasal: Elongate anteroposterior plate or blocky (0); anteroposteriorly compressed into a nearly vertical plate with fossa on ventrolateral surface for posterior nasal sac (caudal sac of Cranford et al., 1996) (1); fossa well excavated with boss delimiting dorsal edge (2) (modified from Muizon, 1988; Messenger & McGuire, 1998).

119. Dorsal surface of nasals: Medial portions distinctly higher than lateral portions (0); forms a fairly flat tabular surface (1); medial portions greatly depressed forming a median trough immediately posterior to nasal openings (2); convex but no obvious median trough or elevation (3) (Muizon, 1988, 1991). Character state “2” appears in some ziphiids and also some kentriodontids where it is called the internasal fossa by Muizon (1988). Cannot be scored for taxa where the nasals are reduced to vertical plates and lack an appreciable dorsal surface.

120. Maximum transverse width of both nasals: Very narrow, < 37% of the maximum width of the external bony nares (0); narrow, width between 55% and 89% of the nares width (1); within 10% of the nares width (2); wide, width between 123% and 140% the nares width (3); very wide, between 152% and 160% the nares width (4); extremely wide, width > 188% the width of external bony nares (5).

121. Combined width of posterior edge of nasals: Wide, width > 150% the maximum width of external bony nares (0); subequal to external nares, width between 85% and 135% the nares width (1); narrow, width between 79% and 50% the nares width (2); very narrow, width between 44% and 39% the nares width (3); extremely narrow, width < 31% the nares width (4). The combined width can only be measured where the nasals meet on the midline. In some taxa there is a more posterior point where the width is wider, but the nasals, in such cases, are separated medially by the frontals.

122. Nasal/Frontal suture: Approximately straight transversely (0); frontal has anterior wedge between posterior ends of nasals (1) (Muizon, 1988).

123. Position of posteriormost edge of nasals: Just anterior to or in a transverse line with anterior edge of supraorbital process of frontal (0); in line with anterior half of supraorbital process of frontal or in line with the halfway point, anteroposteriorly, of supraorbital process (1); in line with posterior half of supraorbital process or in line with postorbital process of frontal (2); in line with gap between postorbital process and the anterior tip of zygomatic process of squamosal or in line with the anterior tip of the latter process (3); between anterior the tip of zygomatic process of squamosal and anterior edge of the floor of squamosal fossa or in line with anterior edge of the floor of squamosal fossa (4); posterior to the anterior edge of the floor of the squamosal fossa (5). The length of the nasal is determined by the positions of its anterior and posterior edges; therefore, its length is not scored as a separate character.

124. Height of posterior portions of nasals relative to lateral edge of maxilla: Approximately equal to height of the base of rostrum (see character 58 for a description of measurement used); nasal height between 92% and 139% the rostral height (0); elevated above rostrum, height of nasals between 156% and 203% the rostral height (1); very elevated, height of nasals between 229% and 282% the rostral height (2); extremely elevated, height of nasals between 354% and 420% the rostral height (3); nasals tower above facial part of skull, height of nasals > 548% the rostral height (4) (modified from Heyning, 1989b).

125. Frontals: Lower than nasals (0); same height as nasals (1); higher than nasals (2) (Muizon, 1988). Messenger & McGuire (1998) coded the presence of the frontal protuberance as a separate character; however, taxa with a frontal protuberance always have the frontals higher than the nasals.

126. Frontals posterior to nasals and between the maxillae: Wider than maximum transverse width across nasals (0); same width as nasals (1); narrower than nasals, maxillae expanded medially posterior to the nasals (2) (Muizon, 1988). Cannot be scored for taxa that lack maxillae posterior to the nasal bone.

127. Dorsal exposure of frontals: Fairly flat with separation between right and left frontal obscure (0); frontals are nodular with distinct separating sulcus on midline (1) (Fordyce, 1994).

128. Anterodorsal wall of braincase: Formed by frontal (0); mostly formed by maxilla (1) (Schulte, 1917; Miller, 1923).

129. Supraoccipital: Below frontals and/or nasals, whichever is higher (0); at same level as frontals and/or nasals (1); higher than frontals and/or nasals (2) (modified from Moore, 1968).

130. Maxilla on dorsal surface of skull: Does not contact supraoccipital and/or nuchal crest posteriorly, maxilla clearly separated by frontal and/or parietal (0); contact present, maxilla reaches nuchal crest and/or supraoccipital (1) (Muizon, 1991, 1994; modified from Geisler & Sanders, 2003). In some extant cetaceans the interparietal and parts of the parietals become integrated into the occiput and are posterior to the nuchal crest. Often sutures in this area fuse, thus this character focuses on whether or not the maxilla reaches the nuchal crest, a feature seen in juveniles as well as adults.

131. Temporal crest: Dorsal surface adjacent to crest is nearly horizontal, crest appears to be directed laterally (0); dorsal surface is concave and surface of temporal fossa below crest faces almost entirely laterally, crest appears to be oriented dorsolaterally (1) (Muizon, 1988).

132. Temporal crest: In posterior position, frontal roofs over the anterior third or more of temporal fossa (0); on posterior edge of supraorbital process of frontal (1); lateral end of temporal crest on dorsal surface of supraorbital process (2); entire crest on dorsal surface of supraorbital process (3) (modified from Heyning, 1989b).

133. Roof of temporal fossa: Frontal (0); frontal but with large opening through which maxilla and/or premaxilla is exposed, margins of the window are formed by a frontal ring (1). This can only be scored in taxa that have a posterior position of the temporal crest.

134. Frontal/parietal suture in lateral view: Vertical or slightly angled posteroventrally (0); dorsal portion of suture pointed and extended far anterior so that the anteriormost point of parietal is anterior to the posteriormost point of premaxilla (1) (Miller, 1923).

135. Parietals in dorsal view (unordered): Contact each other on the midline or are separated by an interparietal (0); are in place in the skull roof but are visible only as small triangular areas at edges of the intertemporal constriction, supraoccipital overlaps median portions of parietals and obscures them (1); are completely absent in the skull roof (2); visible only as triangular areas dorsolateral to supraoccipital, supraoccipital does not overlap parietals but separates and contacts them along an irregular suture (3) (Whitmore & Sanders, 1976; Barnes 1990).

136. Interparietal: Present (0); absent or fused so that it is not distinguishable from parietals and frontals (1).

137. Cross-section through intertemporal region, including parietals: Ovoid cross-section with sagittal crest (0); ovoid but sagittal crest absent (1); pinched ventrally and dorsal part expanded laterally, expanded part is rounded-over in cross-section (2); dorsal part is greatly expanded, overhangs more ventral portions, and lateral edge of dorsal surface is a sharp ridge (3).

138. Length of intertemporal region, ventral view of skull roof with basicranium removed: Intertemporal region absent or short, canal in frontals that contained olfactory stem and bulbs is < 10% of the length from posterior edge of dorsal nasal sinuses to posterior edge of skull (0); long, canal is between 18% and 35% of that length (1); very long, canal > 44% of that length (2).

139. Dorsoventral thickness of intertemporal region: Thin, thickness is < 25% the maximum height of skull, as measured from intercondyloid notch to dorsal-most point of the supraoccipital (0); thick, thickness between 30% and 43% of the skull height (1); very thick, thickness > 54% of the skull height (2).

140. Anteriormost point of the supraoccipital, in dorsal view: In transverse line with space between posterior edge of skull and anterior edge of the floor of squamosal fossa (0); in line with space between anterior edge of the floor of squamosal fossa and the anterior tip of zygomatic process of squamosal (1); in line with gap between anterior edge of zygomatic process of squamosal and the anteriormost point along posterior edge of the supraorbital process of frontal (2); in line with supraorbital process of frontal (3); in line with or anterior to anterior edge of supraorbital process of frontal; anterior edge of supraorbital process is taken at its medialmost point (4) (modified from Miller, 1923).

141. Pronounced bulge anterior to alisphenoid exposure in temporal fossa: Absent (0); present (1).

142. Alisphenoid: Broadly exposed laterally in temporal fossa (0); lateral surface is broadly overlapped by parietal so that only a narrow strip on the ventral edge of temporal fossa is visible in lateral view (1). This character is not applicable for taxa that lack a dorsally expanded alisphenoid.

143. Zygomatic process of squamosal: Directed anteriorly (0); directed anterolaterally (1) (Sanders and Barnes, 2002).

144. Dorsal edge of zygomatic process, skull in lateral view: Gently convex dorsally (0); near anterior end there is a distinct dorsal flange or process, flange usually articulates with frontal (1); concave dorsally (2).

145. Emargination of posterior edge of zygomatic process by sternomastoid muscle fossa, skull in lateral view: Absent, posterior edge forms nearly a right angle with dorsal edge of zygomatic process of squamosal (0); slight emargination (1); deep emargination (2).

146. Width of squamosal lateral to exoccipital, skull in posterior view: Narrow, exposed portion of squamosal < 14% the distance between sagittal plane and lateral edge of exoccipital (0); intermediate width, width between 16% and 35% of that distance (1); wide, width between 40% and 55% of that distance (2); very wide, width > 129% the distance between sagittal plane and lateral edge of exoccipital (3).

147. Depth of squamosal fossa: Absent or very shallow, depth of fossa < 52% the horizontal distance from the supramastoid crest to the point above deepest part of squamosal fossa (0); shallow, depth between 55% and 91% that distance (1); deep, depth between 98% and 168% that distance (2); very deep, depth greater than 180% that distance (3) (derived from Barnes, 1985). The depth of the fossa is measured relative to the level of the supramastoid crest (i.e. dorsal edge of the zygomatic process) which forms the lateral border of the fossa.

148. Longitudinal profile of floor of squamosal fossa: Highly sigmoidal, concave posteriorly in region of a secondary squamosal fossa (sensu Sanders and Barnes, 2002) but convex anteriorly (0); slightly sigmoidal, posterior part concave but does not form a discrete pit (1); flat (2); convex (3).

149. Floor of squamosal fossa: Same dorsoventral thickness anteriorly and posteriorly (0); thickens posteriorly (1).

150. Squamosal prominence: Absent (0); present, forms a medial projection on the supramastoid crest (1) (Sanders & Barnes, 2002).

151. Ventral edge of zygomatic process of squamosal in lateral view: Concave ventrally (0); straight (1); convex ventrally (2).

152. Postglenoid process in lateral view: Tapers ventrally to a point (0); anterior and posterior sides nearly parallel with squared-off ventral end (1); same as state “1” except anteroposterior diameter of postglenoid process is very wide (2).

153. Anterior edge of supraoccipital in dorsoposterior view: Triangular, pointed anteriorly (0); semicircular (1); rectangular (2) (Barnes, 1985).

154. Lambdoidal crests of supraoccipital: Horizontal and directed laterally, overhanging temporal fossae (0); directed dorsolaterally, not or only slightly overhanging temporal fossae (1); very low and not directed either way (2). This crest is in a region where it can also be called the temporal crest or the nuchal crest (see Mead & Fordyce, 2009).

155. Posteromedial wall of temporal fossa: Visible in dorsal view (0); hidden in dorsal view by lateral edges of supraoccipital (1). Cannot be scored for taxa where the anteriormost point of supraoccipital is posterior to the level of the anterior edge of the floor for the squamosal fossa.

156. Occipital shield: Smoothly convex or concave (0); bears distinct sagittal crest (1) (Sanders & Barnes, 2002).

157. Dorsal condyloid fossa: Absent (0); present, situated anterodorsal to dorsal edge of condyle (1); present and forms a deep pit (2) (Sanders & Barnes, 2002).

158. Anterior sinus: Absent (0); present but short (1); elongate with corresponding trough on maxilla (2) (Fraser & Purves, 1960). Inia has a very elongate anterior sinus; however, its corresponding trough on the maxilla is absent. All other taxa that have an elongate sinus also have a well-defined trough.

159. Palatine: Relatively thin, floors posterior part of nasal cavity (0); thick, forms part of the anterior wall of the nasal passages (1) (Miller, 1923).

160. Palatines: Exposed ventrally (0); partially covered by pterygoid dividing it into medial and lateral exposures (1); ventral surfaces covered completely by pterygoids (2) (Miller, 1923; Muizon, 1987).

161. Palatine: Ventral surface flat or convex (0); bears fossa for anterior end of pterygoid sinus (1); fossa well developed, divides palatine into medial and lateral laminae (2) (Muizon, 1988, 1991).

162. Lateral lamina of palatine: Free from or sutured to maxilla (0); fused to maxilla (1) (Muizon, 1988).

163. Pterygoid/palatine suture in ventral view: Angled anterolaterally (0); nearly transverse, pterygoid forms a substantial part of subtemporal crest (1); angled anteromedially (2).

164. Pterygoid sinus fossa: Absent or cannot be distinguished from anterior part of fossa for cavum tympani (0); present, anterior edge approximately in line with anterior edge of foramen ovale (1); present and extended well anterior to foramen ovale (2); extended anterior to anterior edge of orbit (3) (modified from Fraser & Purves, 1960).

165. Lateral lamina (outer plate or external duplication) of pterygoid: Present (0); partial, restricted to region lateral to the hamular process (1); absent (2) (Miller, 1923; Fraser & Purves 1960).

166. Subtemporal crest: Present on alisphenoid and/or pterygoid, marks lateral edge of pterygoid fossa (0); subtemporal crest absent, pterygoid fossa extended laterally into orbital region (1) (derived from Fordyce, 1994).

167. Inferior lamina of pterygoid: Absent or restricted to extreme anterior edge of pterygoid sinus cavity (0); present and floors most of sinus cavity (1) (modified from Fraser & Purves, 1960).

168. Superior lamina of pterygoid: Present and covers most of ventral exposure of alisphenoid (0); absent from the sphenoidal region but present in orbital region (1); partially absent from orbital region (2); completely absent from orbital region (3) (Miller, 1923; Fraser & Purves, 1960).

169. Posterior part of pterygoid sinus fossa, region immediately anterior to exit for mandibular branch of trigeminal nerve: One single fossa (0); split into a smaller and shallower posterior fossa and a much larger anteriorly extended deeper fossa by a low ridge (1); same as state “1” except divided by a high ridge (2).

170. Preorbital lobe of pterygoid sinus: Absent (0); present but small (1); present and enlarged (2); enlarged and forms distinct excavation anterior to optic foramen (3); enlarged and extended posterodorsally over frontals to be roofed by maxilla (4) (Fraser & Purves, 1960).

171. Postorbital lobe of pterygoid sinus: Absent (0); present but small (1); present and enlarged (2); enlarged and forms prominent fossa on ventral surface of supraorbital process of frontal posterior to optic foramen (3) (Fraser & Purves, 1960).

172. Coalescence of pre-orbital and post-orbital lobes of pterygoid sinus dorsal to optic nerve: Absent (0); present (1) (Fraser & Purves, 1960).

173. Hamular process of the pterygoid: Splint like (0); solid, long and subconical (1); hollow and excavated by pterygoid sinus, lateral side highly concave, may or may not have lateral and inferior laminae (2); form thin horizontal plates (3); absent (4) (modified from Fraser & Purves, 1960).

174. Hamular processes of pterygoids: Rounded over in ventral view (0); bear anteroposterior keels (1) (Muizon, 1988).

175. Posteriormost point of hamular process of pterygoid, or medial part of pterygoid if the hamular process is absent: In transverse line with the middle of orbit (0); in line with postorbital process (1); in line with anterior edge of zygomatic process of squamosal (2); in line with middle of zygomatic process (3); in line with the postglenoid process (4).

176. Fossa for pterygoid sinus on alisphenoid posterior to groove for mandibular branch of trigeminal nerve: Absent, bone is flat or not ossified because of enlarged internal foramen ovale (0); shallow fossa (1); deep subcircular fossa (2) (modified from Fordyce, 1994).

177. Falciform process of squamosal: Plate-like with a wide, anteroposteriorly, base (0); rod-like with narrow base (1); poorly-developed or absent (2).

178. Falciform process of the squamosal: Medial surface sutured to lateral lamina of pterygoid (0); not sutured (1). Cannot be scored for taxa that lack a lateral lamina of the pterygoid.

179. Tympanosquamosal recess: Absent (0); absent but a small rectangular fossa for sigmoid process of the tympanic present, its long axis is transverse, and it is located medial to postglenoid process of squamosal (1); present and enlarged, forms a triangular fossa medial and anteromedial to postglenoid process (2); very large, forms large fossa that borders entire medial edge of glenoid fossa (3) (derived from Fraser & Purves, 1960).

180. Lateral edge of middle sinus: Smooth (0); deckle-edged (1) (derived from Fraser & Purves, 1960).

181. Position of alisphenoid/squamosal suture, skull in ventral view: Anterior to external foramen of foramen ovale or homologous groove (0); courses along groove for mandibular branch of trigeminal nerve, or just posterior to it (1); just medial to anterior edge of floor of squamosal fossa, foramen ovale, and/or groove situated entirely on alisphenoid (2).

182. Groove for mandibular branch of trigeminal nerve: Directed laterally and is entirely posterior to pterygoid sinus fossa (0); lateral end of groove wraps laterally around the posterior end of the pterygoid sinus fossa and opens primarily anteriorly (1).

183. Ventral part of squamosal posterior to postmeatic process: Large area of laminated bone, appears externally as multiple sutures (0); small area of laminated bone restricted to ventrolateral edge of squamosal (1); without laminated bone (2) (Kasuya, 1973).

184. Cranial hiatus: Absent, petrosal contacts basioccipital medially or partially separated by narrow fissure (0); present, wide space between basioccipital and both petrosal and squamosal (1); present but constricted, a medial projection of parietal partially divides fenestra (2); absent, parietal contacts basisphenoid and/or basioccipital dividing fenestra in two, an anterior foramen ovale and a large posterior opening (3); absent and posterior opening in state “2” is subdivided into several smaller foramina for nerves and vessels (4) (modified from Heyning, 1989b and Luo & Gingerich, 1999).

185. Periotic fossa: Bowl-shaped (0); has transverse ridge that divides it into anterior and posterior portions (1). Cannot be scored for taxa that lack a periotic fossa.

186. Suprameatal pit of squamosal: Absent (0); present but shallow, situated dorsolateral to spiny process of squamosal (1); forms a deep dorsolateral excavation into squamosal (2).

187. Foramen spinosum: Absent (0); present, located in anteromedial corner of anterior part of periotic fossa near or on squamosal/parietal suture (1) (Muizon, 1994).

188. Posterior portion of periotic fossa: Posteromedial part contains a deep, large fossa (0); fossa present but shallow (1); fossa is highly compressed and forms a narrow slit or a small blind foramen (2); fossa absent, posterior portion of the periotic fossa is of uniform depth (3).

189. Zygomatic process of squamosal: Very short, length of process < 92% of the maximum width of mandibular fossa of squamosal (0); short, length between 103% and 162% of mandibular fossa width (1); intermediate length, length between 171% and 189% of the mandibular fossa width (2); long, length between 198% and 271% of mandibular fossa width (3); very long, length > 300% of the width of mandibular fossa (4). State “3” or state “4” is probably the primitive condition for Cetacea and Artiodactyla; however, the character was coded with state “0” (short zygomatic processes) in order to implement the ordering of states in the phylogenetic analyses.

190. External auditory meatus: Wide (0); narrow (1) (Fordyce, 1994).

191. Vomer: Posterior edge terminates on or at anterior edge of basisphenoid (0); terminates on basioccipital covering basioccipital/basisphenoid suture ventrally (1) (Barnes, 1984).

192. Basioccipital crest: Narrow transversely (0); wide and bulbous (1) (Sanders and Barnes, 2002).

193. Rectus capitus anticus muscle fossa: Absent or poorly developed (0); present with a well-defined anterior edge (1). The anterior edge forms a curved ridge that joins the basioccipital crest laterally and curves posteromedially to join its counterpart at the sagittal plane.

194. Posteroventralmost point of basioccipital crest: Rounded over (0); forms a closely appressed separate flange, a narrow crease separates it dorsally from rest of basioccipital crest (1); distinct flange that projects posteriorly (2); distinct but separated by a pronounced notch that interrupts basioccipital crest (3).

195. Angle formed by the basioccipital crests in ventral view: Parallel with no angle formed (0); 15° to 40° (1); 45° to 68° (2); 74° to 90° (3); > 100° (4) (modified from Muizon, 1991).

196. Hypoglossal foramen: Thick bone separating it from jugular foramen, or jugular notch (0); separating bone very thin or absent, in the latter case hypoglossal foramen becomes confluent with jugular foramen (1).

197. Jugular notch, gap between paroccipital process and basioccipital crest. — Open notch, opening and depth of the notch are roughly equal (0); narrow and almost slit-like, depth is much greater than width of opening (1).

198. Paroccipital process, skull in ventral view: Angled posterolaterally, extends posterior to posteriormost edge of condyle (0); posterior edge in transverse line with posterior edge of condyle (1); posterior edge is well anterior to posterior edge of condyle (2).

199. Tubercule of the malleus: Unreduced (0); highly reduced, almost indistinguishable from articular head (1) (Doran, 1876).

200. Processus muscularis of malleus: Processus muscularis shorter than manubrium of malleus (0); subequal (1); processes muscularis longer than manubrium (2) (modified from Muizon, 1988).

201. Apex of anterior process of the petrosal: In ventral or dorsal view blunt or pointed (0); bears tubercle (1) (Luo & Marsh, 1996).

202. Anterior process in lateral view: Anterior edge of anterior process squared-off (0); comes to a blunt apex (1); comes to a slender point (2) (modified from Muizon, 1988).

203. Apex of anterior process of the petrosal: At same level or dorsal to ventral edge of pars cochlearis (0); well ventral to ventral edge of the pars cochlearis, process appears to be ventrally deflected (1) (modified from Fordyce, 1994).

204. Length of anterior process of the petrosal: Absent (0); present but very short, length < 36% of the length of pars cochlearis (1); short, length between 40% and 94% of the promontorial length (2); nearly the same as length of pars cochlearis, length between 100% and 134% of the promontorial length (3); long, length between 141% and 174% of the promontorial length (4); very long, length > 212% of the length of the pars cochlearis (5) (modified from the following: Muizon, 1988; Luo & Marsh, 1996; Geisler & Luo, 1996). The length of the pars cochlearis was measured from its anterior edge to the ventral edge of the fenestra rotunda.

205. Anterior process in lateral view: Ventral edge convex ventrally or nearly flat (0); ventral edge clearly concave (1) (Muizon, 1988). The fovea epitubaria, which articulates with the accessory ossicle of the tympanic, is greatly expanded anteriorly in state “1”.

206. Anteroexternal sulcus: Absent (0); present on lateral surface of anterior process of petrosal, oriented primarily anteroposteriorly but bowed ventrally (1) (modified from Fordyce, 1994).

207. Sulcus for capsuloparietal emissary vein: Present, forms a dorsoventral groove on lateral side of anterior process immediately anterior to lateral tuberosity (0); absent (1) (derived from Geisler & Luo, 1998).

208. Articulation of anterior process with squamosal: Extensive, most of lateral side contacts squamosal (0); large centrally-oriented ovoid region contacts squamosal, free around the edges (1); contact is very small (2); contact is absent, articulates via ligaments (3).

209. Shape of cross section through anterior process at midlength: Highly elliptical, transverse diameter is < 36% the dorsoventral diameter (0); ovoid, transverse diameter is between 51% and 78% of the dorsoventral diameter (1); approximately circular, transverse diameter between 85% and 134% of the dorsoventral diameter (2); bulbous, transverse diameter > 141% of the dorsoventral diameter (3) (modified from Fordyce, 1994 and Luo & Marsh, 1996).

210. Contact of anterior process of petrosal with portion of ectotympanic bulla anterior to accessory ossicle: Absent (0); present but no clear fossa for articulation on petrosal (1); anterior bullar facet present but shallow with poorly defined medial edge (2); present with well defined medial and lateral edges (3) (Fordyce, 1994). Cannot be scored for taxa in which the bulla is completely fused to the anterior process of the petrosal or in some taxa with very short anterior processes.

211. Flange of anterior process of petrosal: Absent (0); present (1) (Geisler and Luo, 1996; Luo and Marsh, 1996). The flange is here redefined as part of the lateral tuberosity, specifically a horizontal shelf on its lateral and anterior sides that overhangs the anterior process.

212. Lateral tuberosity: Absent (0); present, forms a bulbous prominence lateral to fossa for malleus (1); present and elongate, forms a lateral process that articulates dorsally with squamosal (2) (Muizon, 1991; Luo & Marsh, 1996; Geisler & Luo, 1996).

213. Emargination of lateral edge of petrosal by hiatus epitympanicus: With petrosal in ventral view, emargination is narrow and is situated slightly posterior to base of posterior process (0); emargination is wide and is approximately in line with gap between fenestrae ovalis and rotunda (1).

214. Fossa incudis: Poorly defined or cannot be differentiated from rest of epitympanic recess (0); forms a clear circular fossa (1); circular fossa present on a short pedestal, the incudal process (2) (Luo & Marsh, 1996).

215. Mallear fossa: Present (0); absent or poorly developed (1) (Geisler & Luo, 1996).

216. Ventrolateral ridge of petrosal: Absent (0); present (1); present and expanded (2) (Geisler & Luo, 1996).

217. Lateral side of petrosal: Entire side of petrosal contains pitted and rugose bone (0); all but anterior process is rugose (1); lateral side of posterior process of petrosal is pitted and rugose, remaining portions are smooth (2); entire side of petrosal is smooth (3).

218. Origin of tensor tympani muscle: Deep, pocket-like fossa, may have an anterior groove (0); anterior groove only (1); broad, poorly defined origin without a clear groove (2) (Luo & Marsh, 1996; Geisler & Luo, 1998).

219. Angle between anterior process of petrosal and anterior edge of pars cochlearis: Obtuse, pars cochlearis appears transversely compressed (0); nearly 90 degrees, pars cochlearis looks rectangular or semicircular in ventral view (1); acute, pars cochlearis looks globular (2). Covers part of character 18 of Luo & Marsh (1996).

220. Anteromedial corner of pars cochlearis: Rounded (0); angular (1) (Muizon, 1987; Fordyce, 1994).

221. Pars cochlearis: Most convex part is on ventrolateral surface (0); most convex part is on medial surface. Area of greatest convexity begins anteromedial to fenestra rotunda and extends anterodorsally on the medial face. With petrosal in dorsal view, there is a wide expanse of bone medial to internal acoustic meatus (1).

222. Ridge on anterolateral side of pars cochlearis, petrosal in ventral view: Present and high, forms an anteroposterior ridge that also forms the medial edge of a trough for tensor tympani muscle (0); present and low (1); absent (2) (Luo & Marsh, 1996).

223. Fenestra rotunda: Oval (0); shaped like a teardrop with a fissure directed towards the perilymphatic foramen (1) (Fordyce, 1994).

224. Posterodorsal edge of stapedial muscle fossa: Ventral to or in line with dorsal edge of fenestra rotunda (0); well dorsal to fenestra rotunda (1).

225. Stylomastoid fossa: Absent (0); present, situated on posterior face of pars cochlearis posterodorsal to stapedial muscle fossa (1); enlarged dorsally and medially, covers much of posterior face of pars cochlearis (2); enlarged posterolaterally onto posterior process of the petrosal (3) (Geisler & Luo, 1996).

226. Caudal tympanic process of the petrosal: Prominent, its ventral and posterior edges form a right angle in medial view (0); low, its ventral and posterior edges are joined by a smooth curve (1).

227. Caudal tympanic process of petrosal in posteromedial view: Well separated from crista parotica, no division between stapedial muscle fossa and stylomastoid foramen (0); narrow separation or contact, clear separation of stapedial muscle fossa and stylomastoid foramen (1).

228. Aperture of cochlear aqueduct: Smaller than aperture of vestibular aqueduct (0); approximately the same size (1); much larger with narrow posterior edge (2) (modified from Muizon, 1987 and Fordyce, 1994).

229. Distance between aperture of cochlear aqueduct and fenestra rotunda: No distance, both apertures are confluent (0); narrow, distance < 89% of the distance between fenestra ovalis and fenestra rotunda (1); wide, distance between 96% and 122% of the space between fenestrae ovalis and rotunda (2); very wide, distance > 146% (3) (modified from Geisler & Luo, 1996).

230. Distance between aperture of vestibular aqueduct and fenestra rotunda: Very narrow, distance < 112% of the distance between fenestra ovalis and fenestra rotunda (0); narrow, distance between 121% and 185% (1); wide, distance between 192% and 211% (2); very wide, distance > 222% the distance between fenestra ovalis and fenestra rotunda (3).

231. Elongation of pars cochlearis towards cranial cavity, dorsally and medially: Absent (0); present, inner porous bone expanded towards cranial cavity (1); present, outer periosteal bone of pars cochlearis expanded towards cranial cavity (2) (Geisler & Luo, 1996).

232. Excavation of tegmen tympani at base of anterior process: Absent (0); present, fossa on dorsolateral side of tegmen tympani (1).

233. Dorsal edge of tegmen tympani dorsolateral to internal acoustic meatus and anterior process: Present and high, dorsoventral height > 114% the width of pars cochlearis (0); present, height between 58% and 34% of promontorial width (1); low, height between 23% and 11% (2); forms a low ridge or is absent, height < 4% of the width of pars cochlearis (3) (derived from Fordyce, 1994).

234. Dorsal edge of tegmen tympani lateral to aperture for vestibular aqueduct: Present and very high, dorsoventral height > 112% of the width of pars cochlearis (0); high, height between 95% and 50% that width (1); low, height between 12% and 4% (2); faint ridge (3); absent (4).

235. Fundus of internal acoustic meatus: Funnel-like, smaller at the blind end and wider near the rim (0); tubular (1) (Luo & Marsh, 1996).

236. Lateral wall of internal acoustic meatus: Low, does not protrude noticeably from suprameatal fossa and surrounding bone (0); high, a wedge-shaped area of elevated bone occurs between dorsal edge of tegmen tympani and internal acoustic meatus, extending the latter ventrally and increasing its depth (1).

237. Foramen singulare: In common recess with the spiral cribriform tract, transverse crest separating the foramen singulare from proximal opening of facial nerve canal is well developed (0); in common recess with spiral cribriform tract, transverse crest separating it from proximal opening of facial nerve canal is low, and proximal opening of facial nerve canal within internal acoustic meatus (1); separated by partitions of equal height from spiral cribriform tract and proximal opening of facial nerve canal (2); in common recess with proximal opening of facial nerve canal (3) (modified from Luo & Marsh, 1996; Geisler & Luo, 1996).

238. Proximal opening of facial nerve canal: Anterior to spiral cribriform tract (0); slightly anterior, posterior edge of proximal opening of facial nerve canal is lateral to center of spiral cribriform tract (1); lateral to spiral cribriform tract (2).

239. Morphology of proximal opening of facial nerve canal: Continuous with an anterior fissure (0); oval-shaped (1); circular (2) (Luo & Marsh, 1996).

240. Articular rim: Absent (0); present but small, forms a ridge anterolateral to articulation surface of the posterior process of the petrosal and separated from it by a sulcus; the ridge fits into a corresponding cavity posterolateral and slightly dorsal to spiny process of squamosal (1); present, long, oriented posterodorsally, and posterior end intersects dorsal margin of petrosal; in lateral view has sigmoidal shape (2); present with sigmoidal shape and laterally elongate with hook-like process (3) (modified from Muizon, 1987).

241. Contact of petrosal, not including anterior process, with skull: Distal end of posterior process of petrosal, lateral surface of posterior process of the petrosal, and entire dorsal edge of tegmen tympani (or homologous bone) contact squamosal and possibly parietal (0); same as state “0” except dorsal edge contacts from posterior end of posterior process of petrosal to region just lateral to aperture of vestibular aqueduct (1); only dorsal and lateral sides of posterior process articulate with squamosal (2); petrosal articulates with squamosal along hiatus epitympanicus and adjacent regions on the posterior process (3); petrosal only articulates with skull via ligaments (4).

242. Articulation surfaces on posterior processes of ectotympanic bulla and petrosal: Surfaces smooth (0); bear complimentary longitudinal grooves and ridges (1); fused in adults (2) (Kasuya, 1973; Geisler & Luo, 1996).

243. Ventral surface of posterior process of petrosal, along a straight path perpendicular to its long axis: Concave (0); flat (1); convex (2).

244. Bullar facet on posterior process of petrosal: Restricted to ventral surface (0); extends dorsally onto posteromedial face of posterior process (1) (Fordyce, 1994).

245. Facial nerve sulcus: Long sulcus on posterior process of petrosal or the compound petrosal/tympanic posterior process of most mysticetes (0); short, no sulcus posterior to stylomastoid notch (1) (Luo & Marsh, 1996; Geisler & Luo, 1996).

246. Length of posterior process of petrosal: Absent or very short, length < 47% the length of pars cochlearis (0); short, length between 88% and 119% of the pars cochlearis length (1); slightly longer than pars cochlearis, length between 131% and 153% (2); long, length between 191% and 404% (3); very long, length > 613% of the length of pars cochlearis (4) (modified from the following: Kasuya, 1973 ; Barnes, 1990; Luo & Marsh, 1996). The length of the pars cochlearis is measured as discussed in character 194. The length of the posterior process is measured along its long axis, which is usually directed posterolaterally.

247. Orientation of posterior process of petrosal: Forms an angle < 130° with the long axis of tegmen tympani (0); forms an angle between 135° and 165° (1); directed nearly posteriorly, forms an 180° with tegmen tympani (2) (Kasuya, 1973; Geisler & Luo, 1996).

248. Dorsal edge of posterior process, petrosal in lateral or medial view: Straight or convex ventrally (0); concave ventrally, helps to form the neck of posterior process of petrosal (1) (modified from Geisler & Luo, 1996).

249. Posterior process of petrosal: Robust (0); horizontal plate and very thin for most of its length (1) (Luo & Marsh, 1996).

250. Mastoid exposure of posterior process of petrosal on the outside of skull: Exposed externally (0); not exposed, enclosed by the exoccipital and squamosal (1) (Luo & Marsh, 1996; Geisler & Luo, 1996).

251. Anterior spine, or conical anterior tip, of ectotympanic bulla: Absent (0); present but small (1); present and long (2) (Kasuya, 1973; Muizon, 1987, 1994).

252. Shape of ectotympanic bulla: Narrow and long, width of bulla at sigmoid process is < 64% the length of bulla along its long axis (0); wide, width of bulla > 65% of its long axis (1) (Kasuya, 1973). Following Kasuya (1973) this is determined by comparing the width of the bulla at the sigmoid process to the length of the bulla, measured from anterior end to posterior edge of involucrum.

253. Posterior end of ventromedial keel: Forms a smooth curve around posterior part of involucrum (0); protrudes and points medially (1).

254. Accessory ossicle: Absent (0); present (1) (Luo & Marsh, 1996; Luo, 1998).

255. Accessory ossicle: Small and oblong (0); large and subspherical (1) (Fordyce, 1994; Luo & Marsh, 1996). Cannot be scored for taxa that lack an accessory ossicle.

256. Accessory ossicle or homologous region on lip of bulla: Fused to anterior process of petrosal (0); not fused (1) (Barnes, 1990; Fordyce, 1994; Luo & Marsh, 1996).

257. Lateral furrow of ectotympanic bulla: Present (0); absent (1) (Kasuya, 1973; Messenger & McGuire, 1998).

258. Lateral furrow: Broad sulcus (0); narrow crease (1). Cannot be scored for taxa that lack a lateral furrow.

259. Sigmoid process: Forms a straight transverse plate that is directed perpendicular to long axis of bulla (0); forms a curved plate; proximal part is directed posterolaterally while the distal end curves to point laterally (1) (Kasuya, 1973).

260. Ventral margin of sigmoid process: Present (0); absent; in its place the lateral margin of sigmoid process smoothly turns into a sulcus on lateral side of bulla (1).

261. Dorsal edge of sigmoid process: Contacts sigmoid fossa of squamosal (0); distal end expanded anteriorly to articulate with lateral tuberosity of petrosal only (1); does not articulate with squamosal or petrosal (2) (modified from Luo & Marsh, 1996). This character is related to character eight of Muizon (1988).

262. Elliptical foramen of ectotympanic bulla: Present, connection between ectotympanic bulla and its posterior process is split into two pedicles (0); absent (1) (Kasuya, 1973).

263. Anterior edge of posterior process of ectotympanic bulla: Contacts postmeatic process of squamosal (0); contact absent (1) (Kasuya, 1973).

264. Posterior process of ectotympanic bulla: Contains sporadic areas of laminated bone at posterodorsal end (0); almost entire process is laminated bone (1) (Kasuya, 1973).

265. Thickness of posterior process of ectotympanic bulla: Thick in region ventral to facet for posterior process of petrosal (0); forms a thin lamina (1).

266. Distal end of posterior process of ectotympanic bulla: Thinner or approximately the same thickness as more proximal portions (0); distal end thicker but not hypertrophied (1); hypertrophied in size, forms large nodular mass (2) (Flower, 1872; Kasuya, 1973).

267. Median furrow of ectotympanic bulla: Absent (0); forms an interprominential notch on posterior edge of bulla between inner and outer posterior prominences (1); forms continuous anteroposterior groove on ventral surface of bulla (2) (Kasuya, 1973).

268. Interprominential notch: On posterior side of bulla, it is divided by a transverse ridge originating from the outer posterior prominence (0); transverse ridge is absent (1).

269. Profile of ectotympanic bulla in lateral view: Ventral edge convex or flat (0); concave because of posteroventral expansion of outer posterior prominence (1).

270. Inner posterior prominence: Posterior edge approximately in line with posterior edge of outer posterior prominence (0); posterior edge distinctly anterior to posterior edge of outer posterior prominence (1) (Kasuya, 1973; Muizon, 1987). Degree of posterior extension is determined relative to the long axis of the bulla, not the orientation of the bulla in the skull. This alleviates problems associated with the reorientation of the bulla in the skull.

271. Involucrum: In medial view, dorsal and ventral borders converge anteriorly (0); excavated anterior to base of posterior process so that dorsal and ventral sides are parallel (1) (Fordyce, 1994).

272. Involucrum: Bears prominent transverse groove on dorsal surface that divides involucrum into a thicker posterior part and thinner anterior part (0); groove absent (1).

273. Ridge on inside of bulla: Present, transverse ridge extends laterally from involucrum and partially divides cavum tympani into anterior and posterior portions (0); absent (1).

274. Ventromedial keel of the ectotympanic bulla: Present along entire length (0); terminates approximately at level of lateral furrow (1); poorly defined along entire length (2) (Kasuya, 1973).

275. Shape of ventromedial keel, bulla in dorsomedial view: Nearly straight (0); bowed medially (1).

276. Region on dorsomedial side of ventromedial keel: Flat or convex (0); gently concave (1).

277. Length of skull relative to prelumbar vertebral column: Very short, condylobasal length of skull from 77% to 55% length of cervical plus thoracic portions of vertebral column (0); short, skull length from 100 to 83% length of cervical plus thoracic portions of vertebral column (1); long, 123% to 114% prelumbar column length (2); very long, > 135% prelumbar column length (3) (derived from Miller, 1923).

278. Atlas: Ventral process larger than dorsal process (0); both processes are subequal (1); dorsal process larger (2) (modified from Muizon, 1987, 1988).

279. Atlas and axis vertebrae: Unfused (0); fused together (1).

280. Cervical vertebrae posterior to atlas: All are separate (0); only 2nd and 3rd are fused together (1); 2nd through 4th are fused together (2); 2nd through 5th are fused (3); 2nd through 6th are fused (4); 2nd through 7th are fused (5) (modified from Miller, 1923). Fusion of the cervical vertebrae is related to extreme shortening of the neck.

281. Number of thoracic vertebrae: 18 to 17 (0); 16 to 15 (1); 14 (2); 13 (3); 12 (4); 11 (5); 10 or less (6).

282. Number of thoracic vertebrae with capitular articulations: 11 (0); 10 (1); 9 (2); 8 (3); 7 (4); 6 (5); 4 to 5 (6); 3 or less (7) (Sanders and Barnes, 2002).

283. Capitular articulation facets of the posterior vertebrae: Facets gradually shift downward on sequential vertebrae to fuse with the tubercular facets (0); facets abruptly shift from a position on the neural arch to a pedestal that originates from the centrum on the subsequent vertebra (1) (Flower, 1869; Miller, 1923). Coding modified based on observations in Heyning (1989a).

284. Lateral edge of transverse processes of lumbar vertebrae: Oriented anteroposteriorly (0); angled anteromedially 45° or more, relative to a parasagittal plane (1) (Muizon, 1988).

285. Transverse processes of lumbar vertebrae: Oriented ventrolaterally (0); oriented laterally and horizontally (1) (Sanders & Barnes, 2002).

286. Lumbar vertebrae: Transverse processes narrow distally or are approximately the same anteroposterior width as their bases (0); transverse processes bear greatly expanded distal ends (1) (Muizon, 1988).

287. Centrum of anterior lumbar vertebrae: Short, length < 63% the width (0); long, length between 79% and 136% the width (1); very long, length > 147% the width (2) (Muizon, 1988; Barnes, 1990). The width is measured across the anterior face of the centrum.

288. Number of lumbar vertebrae: One (0); 3 (1); 4 (2); 6 (3); 7 to 8 (4); 9 to 10 (5); 11 to 12 (6); 13 to 16 (7); 16 to 19 (8); > 19 (9).

289. Number of caudal vertebrae: 13 to 15 (0); 16 to 19 (1); 20 to 23 (2); 24 to 27 (3); 27 to 30 (4); 30 to 33 (5); 34 to 60 (6).

290. Ventrolateral processes on manubrium of sternum: Absent (0); present but small, occurs ventral to articulation surface for first costal cartilage or rib (1) (Muizon, 1988).

291. Sternum: Comprised of several bones (0); comprised of one bone (1) (Yablokov, 1965).

292. Sternum: Several ribs attach to sternum (0); one rib attaches to sternum (1) (Messenger & McGuire, 1998).

293. Coracoid process of scapula: Present (0); absent or barely distinguishable from edge of mandibular fossa (1) (Muizon, 1987, 1994).

294. Supraspinous fossa of scapula: Present (0); absent or nearly absent, acromion process on anterior edge of scapula (1) (Muizon, 1987, 1994).

295. Prominent deltoid crest on anterior edge of humerus: Present, forms greatest anteroposterior diameter along shaft (0); forms a knob-like tuberosity (1); tuberosity and crest absent (2) (Sanders & Barnes, 2002).

296. Delto-pectoral tuberosity or farthest anterior point of crest: Closer to proximal head of humerus (0); approximately centered, proximodistally, on shaft (1); closer to distal end of humerus (2) (Muizon, 1988).

297. Radial and ulnar facets of humerus: Forms one articulation surface that is semicircular in lateral view (0); two distinct facets that in lateral view form an obtuse angle (1) (Barnes, 1990).

298. Humerus: Longer than radius and ulna (0); approximately the same length as radius and ulna (1); shorter than radius and ulna (2) (Sanders & Barnes, 2002).

299. Olecranon process: Present as a distinct process (0); present as slightly raised proximal posterior edge (1); absent (2) (Barnes, 1990; Messenger & McGuire, 1998).

300. Manus: Pentadactyl (0); tetradactyl or less digits(1) (Yablokov, 1965).

301. Esophageal forestomach: Present, epithelium is either completely devoid of glands or may contain a small patch (0); absent, first chamber of stomach has glandular epithelium (1) (derived from Mead, 1989; Rice & Wolman, 1990).

302. External throat grooves: Absent (0); one pair, one on each side of midline (1); one to five pairs of grooves (2); more than five pairs (3).

303. Throat grooves: Converge anteriorly (0); parallel (1) (Flower, 1872).

304. Sexual dimorphism: Males 45% larger than females (0); males 14% to 30% larger than females (1); females and males approximately the same size (2); females 5% to 10% larger than males (3); females 20% to 30% larger than males (4) (modified from Yablokov, 1965). Size is based on maximum anteroposterior length of the entire body. Although state 1 or 2 is probably the primitive state for Cetacea, the assignment of states used here simplifies the inclusion of the between state homology assumptions (i.e. ordering the states) in the phylogenetic analyses.

305. Dorsal Fin: Absent (0); dorsal hump (1); present (2) (Messenger & McGuire, 1998).

306. Dorsal margin of mesethmoid: Below level of adjacent premaxilla (0); flush with or nearly flush with premaxilla (1); distinctly above level of adjacent premaxilla (2).

307. Straight line distance between posterior-most point of right premaxilla along opening of bony external nares and the right nasal: Right premaxilla and nasal contact each other or are separated by a narrow gap that is < 15% of the maximum width of the external bony nares (0); intermediate separation, distance is between 17 and 50% of maximum nares width (1); wide, distance is > 60% of maximum nares width (2).

308. Orientation of the medial portion maxilla that is situated on either side of the vertex: Faces mainly laterally (0); faces mainly dorsolaterally (1); faces mainly dorsally (2).

309. Deep wedge of supraoccipital and/or interparietal between frontals on vertex: Absent (0); present (1).

310. Lateral margin of posterolateral sulcus: Low (0); high and as a result posterolateral sulcus is deeply entrenched (1).

311. Deep sagittal sulcus on middle of occiput: Absent (0); present (1).

312. Longest side of nasal faces: Dorsally (0); mainly anterodorsal (1); mainly anteriorly (2).

313. Occipital: Concave (0); flat or nearly so (1); convex (2); bulbous with inflated braincase (3) (Barnes, 1990).

314. Diameter of the largest tooth: Large, maximum tooth diameter ≥ 20% the maximum width of the external bony nares (0); intermediate, 20% nares width > tooth diameter ≥ 10% (1); small, tooth diameter < 10% nares width (2) (modified from Barnes, 1990).

315. Posterior lower teeth: Apex not recurved (0); apex slightly recurved lingually (1); apex strongly recurved lingually (2).

316. At midpoint of rostrum, orientation of porcelaneous part of premaxilla: Faces primarily laterally and slightly dorsally (0); faces dorsolaterally (1); faces mostly dorsally (2); face dorsomedially (3).

317. Premaxillae between anteromedial sulci emanating from premaxillary foramen (prenarial triangle): Convex transversely and rise up towards midline (0); rise up towards midline but concave transversely (1); flat or concave, may slightly rise up towards midline but not noticeably so (2).

318. Canal and/or foramen near posterior end of nasal process of premaxilla near suture with maxilla: Absent (0); present, foramen occurs between the maxilla laterally and the premaxilla medially, lateral or just anterior to the bony external nares, a sulcus continues posteriorly on the maxilla (1); same as sate “1” but the maxilla medial to the sulcus from the foramen is curled over to turn part of the sulcus into a canal (2).

319. Posteromedial sulcus of the premaxilla: Present (0); absent or barely visible (1).

320. Premaxillae anterior to nasal openings: Are flat or concave anteroposteriorly, form a premaxillary sac fossa (spiracular plate) (0); convex anteroposteriorly (1).

321. Anterolateral corner of nasal: Lacks a distinct process (0); bears a thin process that extends anteriorly but is not inflated (1); has a distinct, inflated process that tapers in width anteriorly and ventrally, in lateral view, the angle between the dorsal and anterior face of the process is rounded over (2); same as state “2” but in anterior view, there is a crease that is oriented dorsomedially and separates the distinct process from the rest of the nasal (3). In taxa with nasals that roof of the nasal cavity, the crease would be on the ventral surface of the nasal, although so far it has only been observed in taxa high highly reduced nasals.

322. Parabullary ridge of anterior process: Absent (0); present, occurs on ventral margin of lateral side of anterior process, courses anteroposteriorly, and bulges laterally. Unlike the ventrolateral ridge of mysticetes, this ridge projects laterally not ventrally (1).

323. Posterior part of suture between nasals. —Fairly flat (0); marked by a deep cleft (1) (Muizon, 1988).

324. Medial half of premaxillary sac fossa: Distinctly more excavated than lateral half and surface faces dorsomedially (0); either at same level as lateral half or fairly flat so that it primarily faces dorsally, extreme medial margin may be slightly upturned (1); ascends to form a prominent, longitudinal ridge (2).

325. Postnarial Fossa: Absent (0); present but shallow and undivided (1); present, deep, and subdivided by a median partition (2) (Geisler et al, 2014).

326. Anterior wall of braincase—Posterior to or aligned with posterior margins of supraorbital processes of frontals (0); well anterior to posterior margins of the supraorbital processes of the frontals (1) (Sanders and Geisler, 2015).

327. Tip of snout in lateral view: fairly straight (0); palate is convex but dorsal border is fairly straight in lateral view (1); anterior end of rostrum is upturned with convex palate and concave dorsal border (2).

**328.** **Mesial denticles on main upper molars: none (0); one (1); two (2); three (3); four (4); five (5); six (6). The ultimate maxillary tooth is ignored if it varies substantially from preceding teeth. (Modified from Boessenecker et al., 2020).**

**329.** **Distal denticles on main upper molars: none (0); one (1); two (2); three (3); four (4); five (5); six (6). The ultimate maxillary tooth is ignored if it varies substantially from preceding teeth. (Modified from Boessenecker et al., 2020).**

**330.** **Mesial denticles on main lower molars: none (0); one (1); two (2); three (3); four (4); five (5); six (6). (New).**

**331.** **Distal denticles on main lower molars: none (0); one (1); two (2); three (3); four (4); five (5); six (6). (New).**

332. Posterior teeth with lingual cusps: Absent (0); present (1).

333. Maxillary toothrow, portion of toothrow with multicuspate teeth: long, >50% of maxillary toothrow with accessory cusps (0); intermediate, 50-35% of maxillary toothrow with accessory cusps (1); short, 36-18% of maxillary toothrow with accessory cusps (2); very short, <18% of maxillary toothrow with accessory cusps (3).

334. Embrasure pits: Present (0); absent (1).

335. Bizygomatic width: very small, bizygomatic width <185mm (0); small, bizygomatic width between 185-245mm (1); medium, bizygomatic width between 246-377mm (2); large, bizygomatic width between 378-500mm (3); very large, bizygomatic width between 500-890mm (4); gigantic, bizygomatic width >890mm.

336. Relationship of first two upper incisors: I1 anterior to I2 (0); I2 dorsal to I1 (1).

**337. Transverse cleft on apex of zygomatic process of squamosal: absent (0); present (1). (New).**

**338. Thyrohyoid/thyrohyal: tubular, not flattened, not fused to basihyal; flattened and plate-like, can be fused with basihyal (1). (New).**

**References**

Albright III, L. B., A. E. Sanders, and J. H. Geisler. 2018. An unexpectedly derived odontocete from the Ashley Formatio (upper Rupelian) of South Carolina, U.S.A. Journal of Vertebrate Paleontology 38(4):e1482555.

Allen, G. M. 1921. A new fossil cetacean. Bulletin of the Museum of Comparative Zoology at Harvard College 65:1–14.

Barnes, L. G. 1984. Whales, dolphins and porpoises: origin and evolution of the Cetacea. Series in Geology, Notes for Short Courses 8:139–154.

Barnes, L. G. 1985. Fossil pontoporiid dolphins (Mammalia: Cetacea) from the Pacific coast of North America. Natural History Museum of Los Angeles County Contributions in Science 363:1–34.

Barnes, L. G. 1990. The fossil record and evolutionary relationships of the genus *Tursiops*; pp. 3–26 in S. Leatherwood and R. R. Reeves (eds.), The Bottlenose Dolphin. Academic Press, New York.

Boessenecker, R. W., M. Churchill, E. A. Buchholtz, B. L. Beatty, and J. H. Geisler. 2020. Convergent evolution of swimming adaptations in modern whales revealed by a large macrophagous dolphin from the Oligocene of South Carolina. Current Biology 30:3267–3273

Cranford, T. W., M. Amundin, and K. S. Norris. 1996. Functional morphology and homology in the odontocete nasal complex: implications for sound generation. Journal of Morphology 228:223–285.

Doran, A. H. G. 1876. Morphology of the mammalian ossicula auditus. Transaction of the Linnean Society, Series 2, Zoology 1:371–497.

Fitzgerald, E. M. G. 2006. A bizarre new toothed mysticete (Cetacea) from Australia and the early evolution of baleen whales. Proceedings of the Royal Society B 273:2955–2963.

Flower, W. H. 1867. Description of the skeleton of *Inia geoffensis* and the skull of *Pontoporia blainvilii*, with remarks on the systematic position on these animals in the order Cetacea. Transactions of the Zoological Society of London 6:87–116.

Flower, W. H. 1869. On the osteology of the cachalot or sperm-whale (*Physeter macrocephalus*). Transactions of the Zoological Society of London 6:309–372.

Flower, W. H. 1872. On the recent ziphioid whales, with a description of the skeleton of *Berardius arnouxi*. Transactions of the Zoological Society of London 8:203–234.

Fordyce, R. E. 1994. *Waipatia maerewhenua*, a new genus and species (Waipatiidae, new family), an archaic late Oligocene dolphin (Cetacea: Odontoceti: Platanistoidea) from New Zealand. Proceedings of the San Diego Society of Natural History 29:147–176.

Fordyce, R. E. 2002. *Simocetus rayi* (Odontoceti: Simocetidae, New Family): a bizarre new archaic Oligocene dolphin from the Eastern Pacific. Smithsonian Contributions to Paleobiology 93:185–222.

Fraser, F. C., and P. E. Purves. 1960. Hearing in cetaceans: evolution of the accessory air sacs and the structure of the outer and middle ear in recent cetaceans. Bulletin of the British Museum of Natural History (Zoology) 7:1–140.

Geisler, J. H., and Z. Luo. 1996. The petrosal and inner ear of *Herpetocetus* sp. (Mammalia: Cetacea) and their implications for the phylogeny and hearing of archaic mysticetes. Journal of Paleontology 70:1045–1066.

Geisler, J. H., and Z. Luo. 1998. Relationships of Cetacea to terrestrial ungulates and the evolution of cranial vasculature in Cete; pp. 163–212, in J. G. M. Thewissen (ed.), The Emergence of Whales. Plenum, New York.

Geisler, J. H., and A. E. Sanders. 2003. Morphological evidence for the phylogeny of Cetacea. Journal of Mammalian Evolution 10:23–129.

Geisler, J. H., M. W. Colbert, and J. L. Carew. 2014. A new fossil species supports an early origin for toothed whale echolocation. Nature 508:383–386.

Heyning, J. E. 1989a. Comparative facial anatomy of beaked whales (Ziphiidae) and a systematic revision among the families of extant Odontoceti. Natural History Museum of Los Angeles County Contributions to Science 405:1–64.

Heyning, J. E. 1989b. Cuvier’s beaked whale *Ziphius cavirostris* G. Cuvier; pp. 289–308 in S. H. Ridgway and R. Harrison (eds.), Handbook of Marine Mammals, Vol. 4. Academic Press, New York.

Heyning, J. E., and J. G. Mead. 1990. Evolution of the nasal anatomy of cetaceans; pp. 67–79 in J. Thomas and R. Kastelein (eds.), Sensory Abilities of Cetaceans. Plenum Press, New York.

Kasuya, T. 1973. Systematic consideration of recent toothed whales based on morphology of tympano-periotic bone. Scientific Reports of the Whale Research Institute 25:1–103.

Kellogg, A. R. 1923a. Description of an apparently new toothed cetacean from South Carolina Smithsonian Miscellaneous Collections 76:1–7.

Kellogg, A. R. 1923b. Description of two squalodonts recently discovered in the Calvert Cliffs, Maryland; and notes on the shark-toothed cetaceans. Proceedings of the U.S. National Museum 62:1–69.

Luo, Z. 1998. Homology and transformation of cetacean ectotympanic structures; pp. 269–301 in J. G. M. Thewissen (ed.), The Emergence of Whales. Plenum, New York.

Luo, Z., and P. D. Gingerich. 1999. Terrestrial Mesonychia to aquatic Cetacea: transformation of the basicranium and evolution of hearing in whales. University of Michigan Papers on Paleontology 31:1–98.

Luo, Z., and K. Marsh. 1996. Petrosal (periotic) and inner ear of a Pliocene kogiine whale (Kogiinae, Odontoceti): implications on relationships and hearing evolution of toothed whales. Journal of Vertebrate Paleontology 16:328–348.

McLeod, S. A., F. C. Whitmore, Jr., and L. G. Barnes. 1993. Evolutionary relationships and classification; pp. 45–70 in J. J. Burns, J. J. Montague, and C. J. Cowles (eds.), The Bowhead Whale. Society for Marine Mammalogy Special Publication No. 2.

Mead, J. G. 1975. Anatomy of the external nasal passage and facial complex in the Delphinidae (Mammalia: Cetacea). Smithsonian Contributions to Zoology 207:1–72.

Mead, J. G. 1989. Sheperd’s beaked whale *Tasmacetus shepherdi* Oliver, 1937; pp. 309–320 in S. H. Ridgway and R. Harrison (eds.), Handbook of Marine Mammals, Vol. 4. Academic Press, New York.

Mead, J. G., and R. E. Fordyce. 2009. The therian skull: a lexicon with emphasis on the odontocetes. Smithsonian Contributions to Zoology 627:1–248.

Messenger, S. L., and J. A. McGuire. 1998. Morphology, molecules, and the phylogenetics of cetaceans. Systematic Biology 47:90–124.

Miller, G. S., Jr. 1923. The telescoping of the cetacean skull. Smithsonian Miscellaneous Collections 75:1–55.

Moore, J. C. 1968. Relationships among the living genera of beaked whales. Fieldiana Zoology 53:209–298.

Muizon, C. de. 1984. Les vertébrés fossiles de la Formation Pisco (Pérou). deuxiéme partie: les Odontocétes (Cetacea, Mammalia) du Pliocéne inférieur de Sud-Sacaco. Travaux de l’Institut français d’Études Andines 27:1-188.

Muizon, C. de. 1987. The affinities of *Notocetus vanbenedeni*, an early Miocene platanistoid (Cetacea, Mammalia) from Patagonia, southern Argentina. American Museum Novitates 2904:1–27.

Muizon, C. de. 1988. Les relations phylogenetiques des Delphinida (Cetacea, Mammalia). Annales de Paléontologie 74:159–227.

Muizon, C. de. 1991. A new Ziphiidae (Cetacea) from the early Miocene of Washington State (USA) and phylogenetic analysis of the major groups of odontocetes. Bulletin du Muséum National d’Histoire Naturelle 12:279–326.

Muizon, C. de. 1994. Are the squalodonts related to the platanistoids? Proceedings of the San Diego Society of Natural History 29:135–146.

Rice, D. W., and A. W. Wolman. 1990. The stomach of *Kogia breviceps*. Journal of Mammalogy 71:242–246.

Sanders, A. E., and L. G. Barnes. 2002. Paleontology of the Late Oligocene Ashley and Chandler Bridge formations of South Carolina, 3: Eomysticetidae, a new family of Oligocene Mysticetes (Mammalia: Cetacea). Smithsonian Contributions to Paleobiology 93:313–356.

Sanders, A. E., and J. H. Geisler. 2015. A new basal odontocete from the upper Rupelian of South Carolina, U.S.A., with contributions to the systematics of *Xenorophus* and *Mirocetus*(Mammalia, Cetacea). Journal of Vertebrate Paleontology 35:e890107.

Whitmore, F. C., Jr., and A. E. Sanders. 1976. Review of the Oligocene Cetacea. Systematic Zoology 25:304–320.

Yablokov, A. 1965. Convergence or parallelism in the evolution of cetaceans. International Geology Review 7:1461–1468.

Zhou, K. 1982. Classification and phylogeny of the superfamily Platanistoidea, with notes on evidence of the monophyly of the Cetacea. Scientific Reports of the Whales Research Institute Tokyo 34:93–108.
