# Supplementary figures and images for: New heterodont odontocetes from the Oligocene Pysht Formation in Washington State, U.S.A., and a reevaluation of Simocetidae (Cetacea, Odontoceti)
Source: PeerJ. 2023 Jun 23;11:e15576. doi: 10.7717/peerj.15576 (PMC10292202; doi:10.7717/peerj.15576)

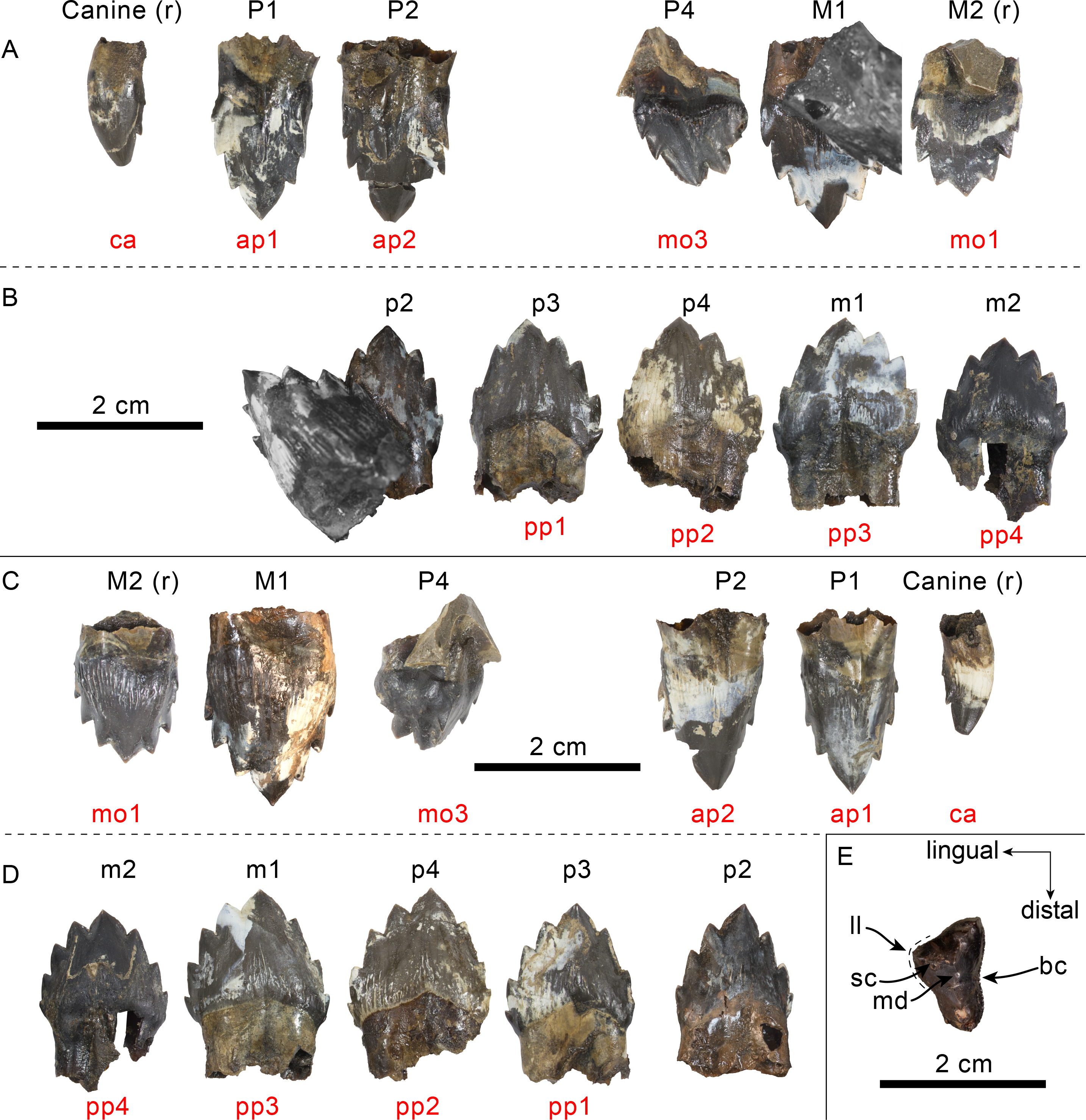

Supplement: Supplemental Information 3 — Upper and lower teeth in buccal (A-B) and lingual (C-D) views; occlusal (E) view of left P4. Tooth positions as interpreted here are shown above each tooth; positions designated in Velez-Juarbe (2017) are shown below (in red). The canine and M2 upper right teeth, reversed for ease of comparison. Teeth labeled as ‘M1’ and ‘p2’ could not be separated from each other during preparation; they were not previously illustrated in Velez-Juarbe (2017). Abbreviations: bc, buccal cingulum; ll, lingual lobe; md, main denticle; r, right tooth, reversed; sc, secondary carina. [file peerj-11-15576-s003.png]

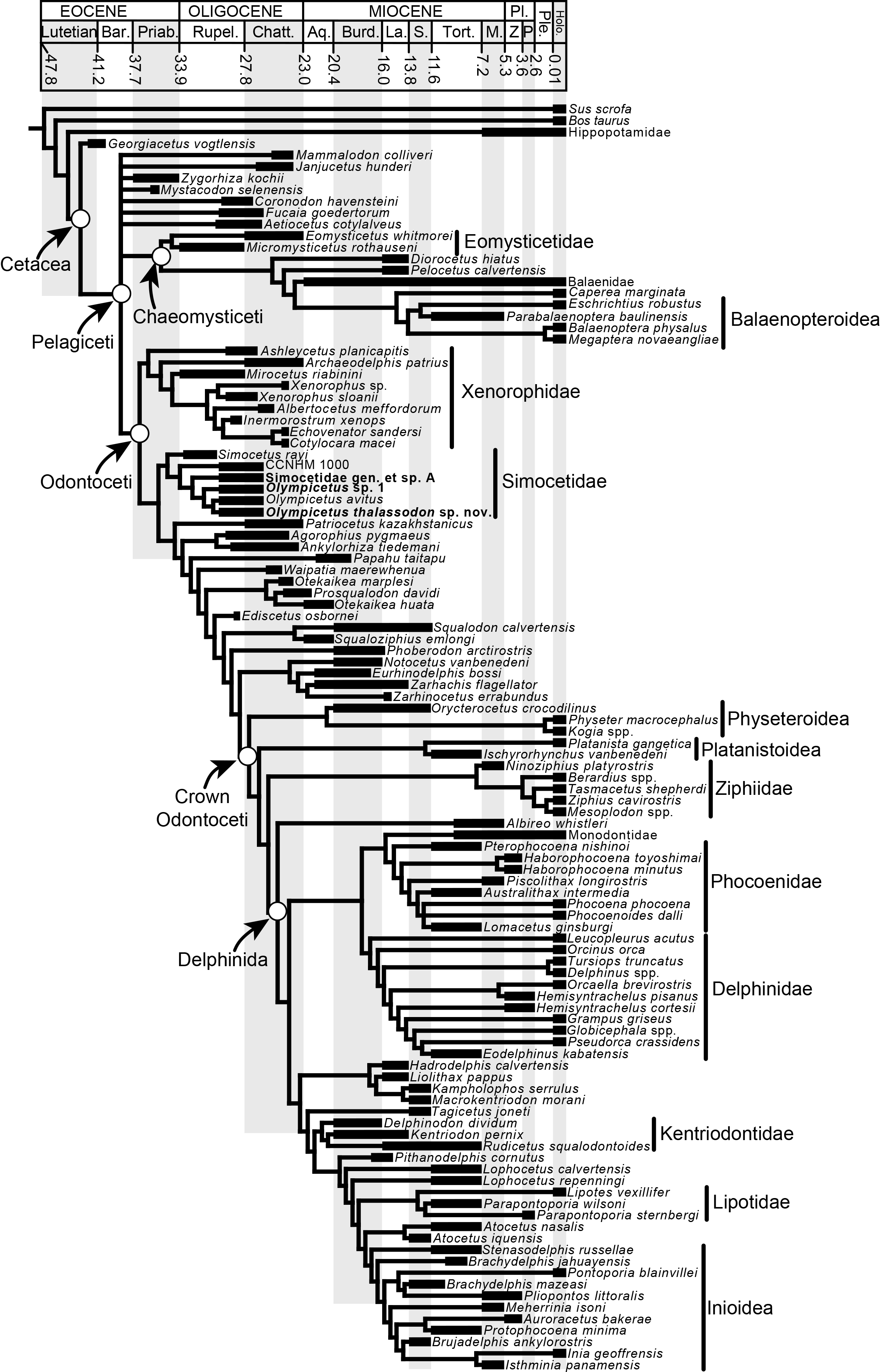

Supplement: Supplemental Information 4 — Phylogenetic tree showing relationship between Simocetidae with other odontocetes. Strict consensus tree based on four most parsimonious trees of length = 3,691, with retention index (RI) = 0.518, and consistency index (CI) = 0.181. Temporal ranges for taxa follow Lloyd & Slater (2021) and Sander et al. (2021). Abbreviations: Aq., Aquitanian; Bar., Bartonian; Burd., Burdigalian; Chatt., Chattian; Holo., Holocene; La., Langhian; M., Messinian; P, Piacenzian; P., Pliocene; Ple., Pleistocene; Priab., Priabonian; Rupel., Rupelian; S., Serravalian; Tort., Tortonian; Z, Zanclean. Time scale based on Cohen et al. (2013). [file peerj-11-15576-s004.png]
